# Genetic basis for the establishment of endosymbiosis in *Paramecium*

**DOI:** 10.1038/s41396-018-0341-4

**Published:** 2019-01-15

**Authors:** Ming He, Jinfeng Wang, Xinpeng Fan, Xiaohui Liu, Wenyu Shi, Ning Huang, Fangqing Zhao, Miao Miao

**Affiliations:** 1 Savaid Medical School, University of Chinese Academy of Sciences, Beijing, 100049 China; 2 Beijing Institutes of Life Science, Chinese Academy of Sciences, Beijing, 100101 China; 3 School of Life Sciences, East China Normal University, Shanghai, 200241 China; 4 Center for Excellence in Animal Evolution and Genetics, Chinese Academy of Sciences, Kunming, 650223 China

**Keywords:** Sequencing, Genomics

## Abstract

The single-celled ciliate *Paramecium bursaria* is an indispensable model for investigating endosymbiosis between protists and green-algal symbionts. To elucidate the mechanism of this type of endosymbiosis, we combined PacBio and Illumina sequencing to assemble a high-quality and near-complete macronuclear genome of *P. bursaria*. The genomic characteristics and phylogenetic analyses indicate that *P. bursaria* is the basal clade of the *Paramecium* genus. Through comparative genomic analyses with its close relatives, we found that *P. bursaria* encodes more genes related to nitrogen metabolism and mineral absorption, but encodes fewer genes involved in oxygen binding and N-glycan biosynthesis. A comparison of the transcriptomic profiles between *P. bursaria* with and without endosymbiotic *Chlorella* showed differential expression of a wide range of metabolic genes. We selected 32 most differentially expressed genes to perform RNA interference experiment in *P. bursaria*, and found that *P. bursaria* can regulate the abundance of their symbionts through glutamine supply. This study provides novel insights into *Paramecium* evolution and will extend our knowledge of the molecular mechanism for the induction of endosymbiosis between *P. bursaria* and green algae.

## Introduction

Endosymbiosis is a widely accepted theory that explains the origin of eukaryotic organelles, such as chloroplasts and mitochondria. Many interesting endosymbiotic events provide insight into horizontal gene transfer and coevolution. For example, two *Hydra* species (*H. viridissima* and *H. vulgaris*) can establish an endosymbiotic relationship with green algae [1, 2]. Gene expression analysis revealed that hosts’ genes associated with oxidative stress can benefit the survival and life cycles of *Hydra*. In addition, biotrophic transport of algal maltose to hosts has been observed, and endosymbiotic algae can also make use of amino acids provided by the *Hydra* host [3], suggesting that the endosymbiotic relationship between hydra and green algae is mutualistic. In another example, the marine mollusk *Elysia chlorotica* exhibits endosymbiosis by acquiring chloroplasts from *Vaucheria litorea* [4, 5]. When *V. litorea* is ingested, all of its cellular components except for the chloroplasts are digested or discarded, whereas the retained chloroplasts could help the host survive for weeks to months under extreme conditions in which only carbon dioxide and light are supplied [5].

**Fig. 1:**
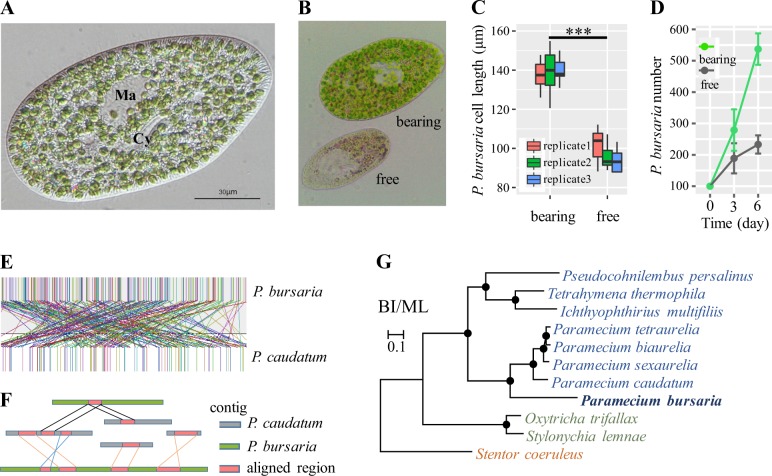
Difference between algae-bearing and algae-free *P. bursaria* and comparative genomic analysis. **a** Differential interference contrast microscope image of a typical *P. bursaria* cell. Ma, macronucleus; Cy, cytopharynx. **b** Microscope images of algae-bearing and algae-free *P. bursaria* are shown. **c** The cell length distribution of algae-bearing and algae-free *P. bursaria* for three independent biological replicates, n = 5. ***P < 0.001, based on a *t*-test. The top and bottom of the box represent the 3rd quartile and 1st quartile, respectively. The band within the box represents the median. **d** The cell proliferation of algae-bearing and algae-free *P. bursaria* within 6 days, feeding with *E. coli* HT115. The original *P. bursaria* cell number is 100, with each curve and error bar representing the mean ± standard deviation from three experimental replicates, respectively. **e** Extremely programmed genome rearrangements between *P. bursaria* and *P. caudatum*. Vertical bars represent collinear blocks of orthologous regions. The collinear blocks between the two genomes are connected by colored lines. **f** A schematic visualization of genome rearrangement between *P. bursaria* and *P. caudatum*, in which multiple inversion and translocation events are present among these contigs. **g** BI and ML trees for 69 orthologous genes of 11 ciliates with the evolutionary model ‘LG+I+G+F’. The solid black dots at nodes indicate Bayesian posterior probabilities (PPs) of 1.0 and bootstrap support (BS) values of 100%

*Paramecium* species, such as *P. bursaria*, provide an excellent opportunity to examine the formation of endosymbiosis because they harbor hundreds of endosymbiotic *Chlorella variabilis* in its cytoplasm (Fig. 1a). Previous studies have reported that the symbiotic algae are enveloped by a perialgal vacuole (PV) membrane beneath the cell cortex (Supplementary Figure S1A), which prevents the algae from being fused by the host’s lysosome [6, 7]. PV-coated algae usually occupy the position of trichocysts under the cell cortex (Supplementary Figure S1B) [6]. The symbiotic *C. variabilis* can divide within the PV membrane, and the division furrow was observed (Supplementary Figure S1C), suggesting that *C. variabilis* in the cytoplasm can not only survive but also proliferate with their host cell’s growth. Furthermore, the newborn algae were also observed crumbling away from their mother cytoderm fragment (Supplementary Figure S1D) [8]. As a result, *P. bursaria* and *C. variabilis* form a relatively stable endosymbiotic relationship under the protection of the PV membrane. Previous study revealed that *P. bursaria* could control the abundance of algal symbionts according to light intensity and gain more benefits from the system, which is a general evolutionary strategy to maintain the stable endosymbiosis within protists [9].

To investigate the contribution of *C. variabilis* to *P. bursaria*, we generated *P. bursaria* without *C. variabilis* symbionts. Since cycloheximide can induce the swelling of the PV and then cause the digestion of PV-free *Chlorella* [10–12], algae-bearing *P. bursaria* was treated with 10 μg/ml cycloheximide for several weeks to generate algae-free *P. bursaria* (Fig. 1b). The length of algae-bearing *P. bursaria* cells was significantly longer than that of cells without algae (*t*-test, *P* < 0.001) (Fig. 1c) [10]. Furthermore, the proliferation of algae-bearing *P. bursaria* was much faster than that of algae-free *P. bursaria* (Fig. 1d), feeding with *E. coli* HT115, suggesting that endosymbiotic algae were vital for *P. bursaria*’s growth and proliferation. Before this experiment, we have utilized filter membrane, penicillin and streptomycin antibiotics and starving methods to eliminate unspecified bacteria.

To better understand their symbiotic relationship, we sequenced the genome and transcriptome of *P. bursaria* and compared them with those of other *Paramecium* species. We constructed one 16–20 Kb DNA library for the PacBio RS II System and generated 721,593 long reads (average sub-read length of 8.6 kb) with an ultra-high sequencing depth (177×). We performed genome assembly using Canu [13] including error correction, read trimming and sequence assembly, which is specifically designed for assembling high-noise, single-molecule sequencing reads. Considering the high error rate of PacBio sequencing, two additional short-fragment libraries (180 and 500 bp) were constructed and sequenced on an Illumina HiSeq 2500 sequencer, which generated 27,378,470 (5.4 Gb) and 32,475,823 (6.4 Gb) PE100 reads, respectively (Supplementary Table S1). We used these two data sets to correct substitution and indel errors in the PacBio assembly using Pilon [14]. Finally, we obtained a 29.2 Mb *P. bursaria* genome, consisting of 405 contigs with no gaps (Supplementary Table S2). Its genome size is much smaller than that of other ciliates but comparable to that of *P. caudatum*. Genomic synteny analysis revealed that *P. bursaria* exhibits tremendous genomic rearrangements compared with *P. caudatum* (Fig. 1e, f). We further constructed a TruSeq Synthetic Long-Read DNA library to generate long reads and verify the accuracy of the assembled genome. In total, 51 Gb TruSeq Synthetic long reads were generated and then assembled into 22,218 long sequences (average length of 7.1 kb). All these synthetic long sequences were aligned to the 29.2 Mb genome using BLASTN (e-value 1e−5). Approximately 96.56% of the reads could be continuously aligned to the assembled genome and the remaining reads were found to be bacterial or algal origins, indicating its high completeness and accuracy (Supplementary Figure S2A). Meanwhile, we used a widely used approach CEGMA [15] to evaluate the completeness and potential contaminations in assembled ciliate genomes. 94.8% of standard core genes could be identified in the assembled *P. burasia* genome, which is similar to the completeness rate of three well-studied ciliate genomes, *T. thermophila* (89.9%), *O. trifallax* (93.1%) and *P. tetraurelia* (92.7%). In addition, we plotted the distribution of GC contents of the assembled contigs and found that they follow a typical ciliate %GC distribution and do not contain any obvious algal or bacterial contaminants (Supplementary Figure S2B). The copy number of MAC (macronucleus) in the ciliate cells is generally several hundreds or thousands of times higher than MIC (micronucleus) [16, 17]. Therefore, the distribution of the Illumina sequencing as of assembled contigs was used to exclude the potential contamination of the MIC genome. As shown in Supplementary Figure S2C, only one peak (230×) of sequencing depth could be found, with more than 93.6% of contigs having a sequencing depth >100×. In addition, we further used single-copy protein-coding genes to evaluate the redundancy of assembled contigs to exclude the potential contamination of the MIC genome.

To predict the protein-coding genes of the *P. bursaria* genome, an RNA-Seq library was constructed and sequenced to generate 6,375,138 paired-end reads (Supplementary Table S1). In addition, a published *P. bursaria* RNA-Seq data set [18] (DRR003755) was also used to improve the completeness of gene prediction. We employed a comprehensive strategy to annotate the genome by combining transcriptome-based, homolog-based and ab initio approaches. In all, 17,266 protein-coding genes were predicted in the *P. bursaria* genome (Supplementary Table S2), which is slightly lower than the number of genes predicted in its close relative *P. caudatum* (18,509). *Paramecium tetraurelia*, *Paramecium biaurelia and Paramecium sexaurelia* have undergone whole-genome duplication events [19], such that their genome size and gene number are twice as large those in *P. bursaria* and *P. caudatum* (Supplementary Table S2). The *P. bursaria* genome and the other ten sequenced genomes of ciliates were selected to investigate their phylogenetic relationships (Supplementary Table S3). These 11 species can be classified into three classes: Oligohymenophorea, Spirotrichea, and Heterotrichea. *Stentor coeruleus* [20] in class Heterotrichea was selected as an outgroup. We used the ortho-MCL approach [21] to find orthologous genes from protein-coding gene sets of these ciliate genomes. In total, 69 orthologous single-copy genes with 23,175 amino acid residues were chosen to construct phylogenetic trees. Both the maximum likelihood tree and Bayesian inference tree were constructed and showed similar phylogenetic relationships to those in previous studies [22–24] (Fig. 1g). Notably, *P. bursaria* formed a sister clade with other *Paramecium* species and occupied the earliest diverging branch of *Paramecium*, indicating that *P. bursaria* is the earliest differentiated species among the five *Paramecium* species.

We annotated the predicted genes of *P. bursaria* and *P. caudatum* based on Gene Ontology (GO) terms, and then performed functional enrichment analysis. A Pearson Chi-Square test was used to select differentially enriched GO terms between *P. bursaria* and *P. caudatum*. Differentially enriched GO terms are involved in a variety of biological processes and molecular functions, including oxidation reduction, drug transporter, oxygen binding, and lipid binding (Supplementary Figure S3A and Supplementary Figure S4). The function of the multidrug and toxin extrusion protein (MATE) family (drug transporter) is to eliminate exogenous and endogenous poisonous compounds from both hosts and symbionts [25, 26], which is the consequence of adaptation to endosymbiosis over evolutionary time. The enriched MATE proteins in *P. bursaria* may confer resistance to toxic compounds on the hosts to maintain the symbiotic relationship. In contrast, genes involved in oxygen binding are depleted in *P. bursaria*, especially for globin genes. Globin proteins including heme and globular proteins are widely distributed in plants, animals and microbes and consist of three globin lineages, which can bind and transport oxygen [27, 28]. The comparison among ciliate genomes indicates that the copy numbers of globin genes between the macronucleus and the micronucleus are similar in both *Tetrahymena thermophila* (12 vs. 9) and *Oxytricha trifallax* (5 vs. 6) (Supplementary Figure S3B). However, the globin gene number of *P. bursaria* is far less than that in other *Paramecium* species (2 vs. 21~46). A possible explanation is that endosymbiotic algae may produce enough oxygen through photosynthesis for *P. bursaria* to maintain cellular respiration in mitochondria. Consequently, an abundant oxygen supply may allow *P. bursaria* to encode fewer oxygen binding genes over the course of long-term evolution. By comparing *P. tetraurelia*, *P. biaurelia and P. sexaurelia* to *P. caudatum*, we found that the average globin gene number in the three *Paramecium* species was twice that in *P. caudatum*, which can be attributed to multiple whole-genome duplication events that occurred in these species. To investigate the phylogenetic relationships among these globin genes, 45 globin sequences were retrieved from *P. tetraurelia*, *P. caudatum*, *P. bursaria* and *O. trifallax* after filtering short fragmented sequences (<100 aa) and then they were used to construct a phylogenetic tree. As shown in Supplementary Figure S3C, globin genes from one species exhibited a dispersed distribution instead of being clustered together, indicating that they may be amplified through speciation.

**Fig. 2:**
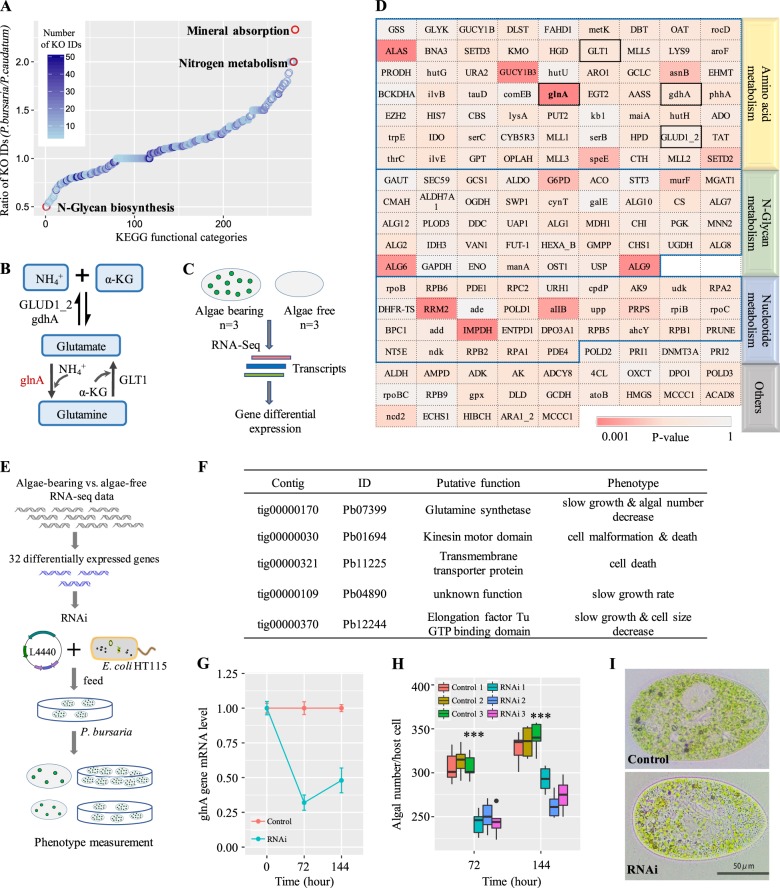
Glutamine biosynthesis contributes to the establishment of endosymbiosis between *P. bursaria* and *C. variabilis*. **a** The ratio of KOs between *P. bursaria* and *P. caudatum* for each KEGG pathway. The ratios for mineral absorption and the nitrogen metabolism pathway are the highest (> = 2). The ratio for the N-glycan biosynthesis pathway is the lowest ( = 0.5). **b** The biosynthetic pathway of glutamine and glutamate. Glutamate dehydrogenase (GLUD1_2) and NADP-specific glutamate dehydrogenase (gdhA) can reversibly catalyze oxidative deamination of glutamate to produce ammonia and alpha-ketoglutarate (α-KG). Glutamine synthetase (glnA) can catalyze ammonia and glutamate to generate glutamine. NADH-dependent glutamate synthase (GLT1) can catalyze one glutamine and one α-KG to generate two glutamates. **c** The flow chart of differential gene expression analysis between algae-bearing and algae-free *P. bursaria* for three independent replicates. **d** Differential expression analysis of 165 genes related to nitrogen metabolism between algae-bearing and algae-free *P. bursaria*. The color depth represents the P value (*t*-test). The most significant differentially expressed gene (glnA, P = 0.0013) is highlighted in a black box. **e**–**i** Functional validation of differentially expressed genes using RNAi. **e** The workflow of the RNAi experiment. A L4440 plasmid with the target gene was transferred into *E. coil* HT115. The phenotype of *P. bursaria* was measured before and after the hosts were fed wit *E. coil* HT115. **f** The list of five genes which exhibited significant phenotype changes after knockdown. **g** The expression level of glnA at 72 and 144 h after RNAi using RT-qPCR with Centrosomal protein gene as an internal reaction control. The curve and error bar represent the mean ± standard deviation for three independent experimental replicates. Control represents *P. bursaria* fed with *E. coil* HT115 including an empty L4440 vector. RNAi represents *P. bursaria* fed with *E. coil* HT115 including the RNAi vector L4440. **h** The algal number per host cell after 72 and 144 h for three independent experimental replicates (n = 5 cells for each replicate). ***P < 0.001, based on a *t*-test. (**i**) Microscope images of *P. bursaria* in the control and RNAi groups. After RNAi, *P. bursaria* harbors fewer algae than that in the control group

We then annotated the predicted protein-coding genes to the KEGG pathway. Compared with those of *P. caudatum*, *P. bursaria* genes exhibited obviously different functional enrichments in a number of KO categories, including mineral absorption, nitrogen metabolism and N-glycan biosynthesis (Fig. 2a). *P. bursaria* encodes fewer genes involved in N-glycan biosynthesis, but instead it has more genes related to mineral absorption and the nitrogen metabolism pathway. The transient receptor potential cation channel subfamily M member 6 (TRPM6) gene of hosts encodes a protein including an ion channel domain and a protein kinase domain, which can transport the Mg^2+^ that plays a key role in harvesting solar energy during photosynthesis [29, 30]. This finding indicates that *P. bursaria* may supply its endosymbiotic algae with Mg^2+^ to ensure the symbionts’ ability to photosynthesize, which may explain the ability of *P. bursaria* to manipulate symbionts load according to light intensity [9]. However, the exact mechanism of how *P. bursaria* hosts supply Mg^2+^ to chlorella is still not fully understood. Previous studies revealed that the number of amino acid transporters in endosymbiotic algae increases significantly, and endosymbionts could obtain amino acids from *P. bursaria* as a nitrogen source [18, 31, 32]. According to the results of KO enrichment analysis, we speculated that nitrogen metabolism, especially glutamine and glutamate biosynthesis (Fig. 2b), may be a critical factor for this endosymbiotic system. To clarify the role of nitrogen metabolism in the endosymbiosis, we conducted differential expression analysis using the transcriptome data sets of *P. bursaria* with and without *C. variabilis* symbionts (Fig. 2c) [18]. Differential expressions of 165 genes related to nitrogen metabolism (amino acid metabolism, N-glycan metabolism and nucleotide metabolism) were explored (Fig. 2d). The glnA gene ranks the first among these differentially expressed genes (*P* = 0.0013), as its expression level in algae-bearing *P. bursaria* is up-regulated four-fold compared to that in algae-free cells (average FPKM 155 vs. 38).

To further investigate the relationship between *P. bursaria* and its symbiotic algae, we used RNAi experiments to knock down 32 most differentially expressed genes using the homology-dependent gene silencing approach (Supplementary Table S4) [33–36]. We firstly cloned these target genes from the cDNA library of *P. bursaria*. The expression vector L4440 with the target gene sequence was induced by IPTG to produce dsRNA, which can knock down the expression of the target gene of the hosts. Subsequently, *P. bursaria* were fed the transformed *E. coli* HT115 consisting of the L4440 plasmid with the target gene under normal culture conditions (Fig. 2e). When *E. coli* HT115 is digested by *P. bursaria*, the dsRNA of target gene will be discharged into the hosts’ cytoplasm and then will be cleaved into short-interfering RNAs (siRNAs) by Dicer [33–35]. Eventually, an RNA-induced silencing complex consisting of the guide strand will specifically bind and cleave the target mRNA in *P. bursaria*. After 6 days, we examined the phenotype of transformed *P. bursaria* by measuring their cell shape and size, growth rate, and endosymbiotic algae number. As shown in Fig. 2f and Supplementary Figure S5, the knockdown of five genes could cause obvious abnormal phenotypes, including smaller cell size, reduced symbiotic algae, slow growth rate or cell depth. Strikingly, the knockdown of Pb07399 (glnA) could significantly reduce the number of symbiotic algae and the growth rate but did not affect the host cell size (Fig. 2g–i and Supplementary Figure S5). We counted the algal number per *P. bursaria* cell using micrographs of each crushed host cell and found that the algal number of the glnA RNAi group was significantly lower than that of the control group (*t*-test, *P* < 0.001) (Fig. 2h, i and Supplementary Figure S6). These findings indicated that *P. bursaria* may supply glutamine for *C. variabilis* as a nitrogen source, and the host cells can regulate the abundance of endosymbiotic algae through the expression of their glnA gene.

**Fig. 3:**
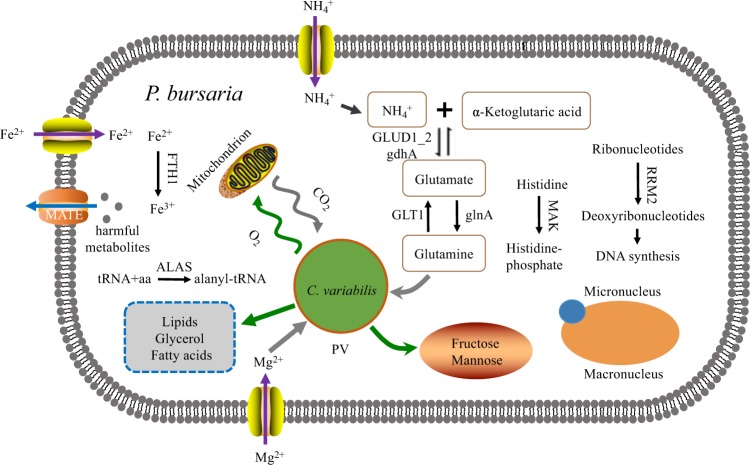
A schematic summary of the metabolic interaction between *P. bursaria* and *C. variabilis*. Green arrows indicate that symbionts provide hosts with O_2_, carbohydrates, and lipids, whereas gray arrows indicate that hosts supply symbionts with Mg^2+^, CO_2_, and glutamine. The blue arrow indicates that the MATE protein family excretes harmful metabolites. Hosts can absorb Fe^2+^, Mg^2+^, and NH_4_^+^ for themselves and for symbionts

Based on the comparative genomic analyses, we propose a model of the symbiotic relationship between *P. bursaria* and its symbiotic algae (Fig. 3). *P. bursaria* can produce glutamine and Mg^2+^ for the symbiotic algae, and the algae can take glutamine as a nitrogen source and utilize Mg^2+^ for chlorophyll-based photosynthesis. In return, symbiotic algae may provide photosynthetic products, such as fructose, maltose, and oxygen, to host cells. Previous studies also proved our findings that there are nutrient trading systems between hosts and symbionts (e.g. O_2_, CO_2_, maltose and amino acids) [37].

The transcriptomes of *P. bursaria* symbiont-bearing and symbiont-free and endosymbiotic *C. variabilis* NC64A genome illustrated some genes correlated with endosymbiosis, such as glutathione S-transferase, 70 kDa HSP (heat shock protein), some amino acid transporters and lipase [18]. Several studies also reported that the abundance of algal symbionts could be regulated by host according to light intensity [9, 18, 31]. In addition, the symbiotic algae are located under the cell cortex which may promote their photosynthetic efficiency. After removing algae from the host cells by using a cycloheximide treatment, the growth of algae-free *P. bursaria* was significantly inhibited and the cells became smaller [10], indicating that symbiotic algae can supply important nutrition for *P. bursaria* growth. Interestingly, the oxygen-binding ability of *P. bursaria* is lower due to the sufficient supply of oxygen from its symbiotic algae. For the host cells, the phototrophic symbionts can produce oxygen to keep their aerobic metabolism stable [38]. The globin gene family also undergoes extensive gene loss during adaptation to oxygen-enriched conditions. Symbiotic algae may produce metabolites that are harmful to their hosts. More MATE genes are present in *P. bursaria*, which help hosts eliminate these harmful metabolites. The RNA interference experiment in this study confirmed the essential role of glutamine in maintaining the endosymbiotic relationship between *P. bursaria* and *C. variabilis*. The algal number per host cell was reduced when the expression level of glnA in *P. bursaria* was down-regulated. As shown in Supplementary Figure S7, the glnA gene in algae-bearing *P. bursaria* exhibited more correlations with other genes than in algae-free *P. bursaria*. When the endosymbiotic system is established in *P. bursaria*, the host encounters more complex conditions and triggers sophisticated regulation of gene expression, including glnA. Previous studies found that glnA mRNA levels and specific activities of glutamine synthetase could be regulated by nitrogen [39]. Through regulation of its glutamine production, *P. bursaria* can farm hundreds of *C. variabilis* to obtain enough oxygen as well as carbohydrates for use as a carbon source.

This study provides a valuable model to examine the mechanism of endosymbiosis as well as a favorable candidate to study the origin of eukaryotic organelles. We believe that this study offers a unique opportunity to research ‘in progress’ genetic changes that are caused by endosymbiosis.

## Materials and methods

### *P. bursaria* culture

*P. bursaria* 110224 was provided by the Institute of Evolution and Marine Biodiversity at the Ocean University of China, collected from Zhongshan Park, Qingdao, China. It was incubated at 25 °C in sterilized distilled water under fluorescent lighting (20–40 μmol photon/m^2^ s) in an incubator. An anatomical lens was used to check the status and count numbers. Differential interference contrast microscope was employed to observe cellular morphological structure [6, 8]. After 7–10 days, approximately 10^5^ *P. bursaria* cells could be harvested per 300 ml of medium. In addition, an algae-free *P. bursaria* was generated by adding cycloheximide (10 μg/ml) for several weeks under normal culture conditions [10, 11]. The *Paramecium* strain and other related materials will be available upon request.

### Nucleic acid preparation

To eliminate bacterial contamination and harvest more *P. bursaria* cells, a 15-μm-pore-size nylon filter membrane was used to isolate the cells from the medium [40]. The resulting cells were washed three times with sterilized distilled water, and the cells were starved and incubated with 1 × penicillin-streptomycin antibiotics (Invitrogen, Carlsbad, USA) for 24 h to further eliminate the bacterial contamination [19, 20]. The treated samples were then centrifuged at 10,000 rpm for 10 min to collect *P. bursaria*. Precipitations of approximately 7 × 10^6^ *P. bursaria* cells were used for DNA and RNA extraction. DNA was extracted using a DNeasy Blood & Tissue Kit (Qiagen, Düsseldorf, Germany) according to the manufacturer’s instructions. RNA was isolated using an RNeasy Mini Kit (Qiagen, Düsseldorf, Germany).

### Genome and transcriptome sequencing

Approximately 15 μg DNA was used to construct a 16–20 kb DNA library according to the guide for preparing a SMRTbell template and sequenced on a PacBio RS II system (Pacific Biosciences, Menlo Park, CA). Additionally, ~10 μg DNA was sheared into ~180 bp and ~500 bp fragments on a Covaris S220 system (Covaris, Woburn, MA). Two DNA libraries with different insert sizes were built using a Nextera DNA Flex Library Prep kit (Illumina, San Diego, CA) and then sequenced (PE100) on an Illumina HiSeq 2500 platform (Illumina, San Diego, CA). Genomic DNA (10 μg) was prepared for a TruSeq Synthetic Long-Read DNA library. The gDNA was fragmented to length of ~10 kb, and long DNA fragments were scattered in 384 wells. Each well with fragments was treated as a small library and sequenced using the HiSeq 2500 system. An RNA library of 150 bp insert size was prepared and sequenced (PE100) using an Illumina HiSeq 2500 platform. Library quality and concentration determination were performed using a Fragment Analyzer (AATI, Ankeny, IA) and a StepOne Plus real-time PCR system (Applied Biosystems, Foster City, CA).

### Genome assembly

High-quality PacBio sub-reads were assembled to a draft genome using Canu software with the ‘genomeSize = 30 M’ parameter [13]. The Canu software includes correction, trimming and assembling steps. Subsequently, PE reads from two short insert-size (180 bp and 500 bp) libraries were imported to Pilon (--genome genome.fasta, --bam input.bam) [14] to correct the draft genome. Assemblies of the TruSeq Synthetic Long-Read DNA library were employed to validate the accuracy of the corrected genome by BLASTN (e-value 1e-5). Finally, we generated a near-complete 29.2 Mb *P. bursaria* genome without gaps, including a mitochondrial genome sequence. Mauve software [41] was used to discover conserved synteny with rearrangements between *P. bursaria* and *P. caudatum*. The genome sequence of *P. bursaria* and its annotation can be accessed at the public database BIGD (http://bigd.big.ac.cn, BioProject accession: PRJCA001086).

### Gene prediction and annotation

To identify protein-coding genes, de novo gene prediction, homolog-based and transcriptome-based methods were combined. GeneMark-ES Suite 4.2.1 (--ES --max_intron 100 --min_gene_prediction 100) [42] and Augustus version 3.2.2 with default parameters [43] were adopted for de novo gene prediction. The protein sequences of four *Paramecium* species (*P. caudatum*, *P. tetraurelia*, *P. sexaurelia*, and *P. biaurelia*) were integrated by using Exonerate version 2.2.0 [44] with the Protein2Genome model (--geneticcode 6 --minintron 10 --maxintron 100, --score 300 --bestn 1) to predict protein-coding genes. The RNA-Seq raw data were trimmed with Trimmomatic [45] to remove adapters and filter low-quality reads (TruSeq3-PE.fa:2:30:10 LEADING:3 TRAILING:3 MINLEN:80 SLIDINGWINDOW:4:15). The trimmed reads were mapped to the *P. bursaria* genome using Tophat2 (-i 10, -I 100) and Cufflink (--min-intron-length 10, -I 100) [46, 47] to generate transcripts and to guide gene prediction. inGAP-CDG [48] was also used for gene prediction from the transcriptomic data sets. All of the gene sets from the three approaches were merged to produce the eventual gene sets.

The non-redundant protein database (NR) and SWISS-PROT database were used to annotate protein-coding genes by BLASTP (e-value 1e−5). The Pfam-A database was used to annotate protein domains by hmmscan using default parameters [49]. The gene name was derived from the best hit. The differential genes were analyzed using KEGG pathway and GO enrichment analyses. KEGG pathway analysis was achieved by using KAAS (KEGG Automatic Annotation Server). Gene ontology (GO) analysis was annotated through InterPro (with default parameters), including the Pfam, PANTHER, SMART, PROSITE, and PRINTS databases. GO enrichment analysis was achieved using Web Gene Ontology Annotation Plot (WEGO) with Pearson Chi-Square test [50]. A published RNA-sequencing data set of *P. bursaria* with and without *C. variabilis* symbionts [18] was utilized to estimate the transcript abundance and to quantify gene expression levels using the Tophat2-Cufflink pipeline (min intron length 10, max intron length 100). The genes with *P* < 0.05 and fold change > 2 were treated as differentially expressed genes. The Pearson correlation values were computed using R and the co-expression network analysis was displayed using Gephi (https://github.com/gephi/gephi).

### Phylogenetic analysis

To identify orthologous genes among *P. bursaria* and ten other published ciliate genomes, the get_homologous software [21] with the MCL algorithm was used. Using MAFFT version 5 [51], we performed multiple alignment for each orthologous gene. Gblocks version 0.91b [52] was used to eliminate poor regions and then select conserved blocks. In totally, 69 orthologous genes with 23,175 amino acid residues were used to conduct the phylogenetic analysis (Supplementary Table S5). ProtTest 3 [53] was employed to select optimum evolutionary model. RAxML [54] constructed the ML tree with the optimum model for tandem orthologous protein sequences (bootstrap 100). A BI phylogenetic tree was constructed for tandem sequences at CIPRES Science Gateway Web (https://cushion3.sdsc.edu/portal2/login!input.action) with the options ‘ngen = 1,000,000, samplefreq = 100 nchains = 4’ and the selected model. The topological structures of the ML and BI phylogenetic trees were compared.

### RNAi experiment

Total RNA of *P. bursaria* was prepared using an RNeasy Mini Kit (Qiagen, Düsseldorf, Germany). Single-stranded cDNA was synthesized with the Promega A5000 reverse transcription system (Promega, Madison, WI) following the user’s guide. Oligo-dT primer was used to reduce bacterial contamination in the samples. The cDNA was provided as the template for PCR and RT-PCR. The PCR product of the target gene was ligated into the pMD19-T vector (Supplementary Figure S8A) and then transferred into JM109-competent cells. Positive clones were selected for plasmid extraction. A double restriction-enzyme digestion segment generated using PstI and KpnI from a pMD19 plasmid was ligated into a L4440 plasmid, which consisted of two T7 promoters (Supplementary Figure S8B). Subsequently, the L4440 plasmid was transferred into HT115-competent cells and spread on a plate (LB medium with tetracycline and ampicillin). Positive clones were cultured and induced by 0.4 mmol/L IPTG to express dsRNA. Cultures (5 ml) were grown to an OD600 value of 1 and then centrifuged at 7000 rpm for 2 min to harvest *E. coil* HT115 with the L4440 plasmid. *E. coil* HT115 from 0.25 ml medium were re-suspended into 0.25 ml ultrapure water and then added into the 2.5 ml RNAi experimental system including ~1000 *P. bursaria* cells, IPTG and ampicillin. Each day we cultured new *E. coil* HT115 and added them into the experimental system. When the *E. coil* cells were digested, dsRNA of the target gene expressed by the L4440 plasmid was discharged into the cytoplasm of hosts to mediate the target mRNA degradation.

## Supplementary information

Suppl. figures and tables
