# Supplementary material for: Genetic basis for the establishment of endosymbiosis in Paramecium
Source: ISME J. 2019 Jan 15;13(5):1360–9. doi: 10.1038/s41396-018-0341-4 (PMC6474222; doi:10.1038/s41396-018-0341-4)
Supplement: Supplementary file 1 — Suppl. figures and tables [file 41396_2018_341_MOESM1_ESM.docx]

**Suppl. Table S1. Sequencing data generated in this study**

| **Data type** | **Library insert size** | **#Reads** | **Data size** | **Sample type** |
| --- | --- | --- | --- | --- |
| Illumina (PE100) | 180 bp | 27,378,470 | 5.4 G | DNA |
| Illumina (PE100) | 500 bp | 32,475,823 | 6.4 G |  |
| TruSeq Long-read (PE100) | 750 bp | 252,144,747 | 51 G |  |
| PacBio | 16-20 kb | 721,593 | 6.2 G |  |
| Illumina (PE100) | 150 bp | 6,375,138 | 1.2 G | RNA |

**Suppl. Table S2. Summary of *Paramecium* genomes**

|  | ***P. bursaria*** | ***P. caudatum*** | ***P. tetraurelia*** | ***P. sexaurelia*** | ***P. biaurelia*** |
| --- | --- | --- | --- | --- | --- |
| Contig | 405 | 777 | 1907 | 543 | 2346 |
| Size (bp) | 29,155,737 | 30,480,878 | 72,065,651 | 68,016,588 | 76,963,137 |
| Coverage | 177x | 186x | BAC | 42x | 42x |
| GC (%) | 28.7 | 28.6 | 28.1 | 24.4 | 26.3 |
| N50 (bp) | 96,293 | 312,922 | 146,932 | 425,007 | 150,218 |
| Average length | 71,812 | 39.229 | 37,790 | 125,261 | 32,806 |
| Max length | 266,355 | 793,585 | 551,073 | 1,303,432 | 1,048,449 |
| Min length | 6,190 | 200 | 668 | 220 | 211 |
| Gene length | 1,588 | 1,445 | 1,431 | 1,460 | 1,456 |
| Exons per gene | 2.9 | 3.5 | 3.6 | 3.3 | 3.6 |
| Average exon (bp) | 537 | 399 | 419 | 379 | 378 |
| Average intron (bp) | 27.6 | 24.7 | 24.2 | 30.3 | 31.4 |
| Gene count | 17,266 | 18,509 | 39,521 | 34,939 | 39,242 |

**Suppl. Table S3. Summary of 10 other ciliate genomes used in this study**

| Species | Genome | | | | | |
| --- | --- | --- | --- | --- | --- | --- |
|  | Size (Mb) | GC% | Gene | Contigs | coverage | Update date |
| *Paramecium tetraurelia* | 72.07 | 28.1 | 39,581 | 1,907 | - | 2008/12/4 |
| *Paramecium biaurelia* | 76.96 | 26.3 | 39,242 | 2,362 | 42× | 2014/7/25 |
| *Paramecium sexaurelia* | 68.02 | 24.4 | 34,939 | 543 | 42× | 2014/7/25 |
| *Paramecium caudatum* | 30.48 | 28.6 | 18,509 | 777 | 186× | 2014/8/21 |
| *Ichthyophthirius multifiliis* | 48.72 | 15.9 | 8,213 | 2,274 | 17× | 2011/7/18 |
| *Stylonychia lemnae* | 54.71 | 32.6 | 20,740 | 52,855 | - | 2012/4/13 |
| *Tetrahymena thermophila* | 104 | 22.1 | 26,996 | 12,923 | 9.08× | 2012/5/10 |
| *Oxytricha trifallax* | 67.17 | 31.4 | 24,983 | 22,363 | 125× | 2013/3/13 |
| *Pseudocohnilembus persalinus* | 55.46 | 18.8 | 13,179 | 288 | 100× | 2015/11/24 |
| *Stentor coeruleus* | 83 | 30.0 | 34,506 | 9,198 | 90x | 2014/5/28 |

| **Suppl. Table S4. Differentially expressed genes (top 32)** | | |  |
| --- | --- | --- | --- |
| Contig | ID | Function | |
| tig00000015 | Pb000015 | Tetratricopeptide repeat | |
| tig00000028 | Pb000028 | Arrestin (or S-antigen), N-terminal domain | |
| tig00000000 | Pb00011 | cGMP-dependent protein kinase interacting domain | |
| tig00000015 | Pb001526 | unknown function | |
| tig00000154 | Pb001547 | Ammonium Transporter Family | |
| tig00000157 | Pb001577 | Replication initiation and membrane attachment | |
| tig00000007 | Pb00517 | Iron-containing redox enzyme | |
| tig00000061 | Pb00635 | Carbohydrate binding module 27 | |
| tig00000047 | Pb00763 | unknown function | |
| tig00000014 | Pb00922 | Ring finger domain | |
| tig00000020 | Pb01297 | ABC transporter | |
| tig00000023 | Pb01411 | unknown function | |
| tig00000030 | Pb01694 | Kinesin motor domain | |
| tig00000032 | Pb01860 | unknown function | |
| tig00000350 | Pb01910 | Perilipin family | |
| tig00000035 | Pb02159 | Anaphase-promoting complex subunit 4 WD40 domain | |
| tig00000101 | Pb04573 | ABC-2 type transporter | |
| tig00000109 | Pb04890 | unknown function | |
| tig00000115 | Pb05232 | Homeodomain-like domain | |
| tig00000134 | Pb06001 | unknown function | |
| tig00000147 | Pb06534 | E1-E2 ATPase | |
| tig00000147 | Pb06580 | IMP dehydrogenase / GMP reductase domain | |
| tig00000157 | Pb07004 | Helix-hairpin-helix containing domain | |
| tig00000170 | Pb07399 | Glutamine synthetase | |
| tig00000211 | Pb08558 | Adenylate and Guanylate cyclase catalytic domain | |
| tig00000268 | Pb10095 | Papain family cysteine protease | |
| tig00000271 | Pb10173 | unknown function | |
| tig00000284 | Pb10465 | unknown function | |
| tig00000321 | Pb11225 | Transmembrane transporter protein | |
| tig00000342 | Pb11758 | Tetratricopeptide repeat | |
| tig00000343 | Pb11778 | unknown function | |
| tig00000370 | Pb12244 | Elongation factor Tu GTP binding domain | |

| **Suppl. Table S5. List of orthologous genes for the phylogenomic analysis (69)** | |
| --- | --- |
| Gene ID | annotation |
| XP_976589.2 | NADPHcytochrome P450 family reductase |
| XP_001025290.2 | kelch repeat protein |
| XP_001023164.2 | peptidyl-prolyl cis-trans isomerase, cyclophilin-type protein |
| XP_001014751.2 | transcription factor/nuclear export subunit protein |
| XP_001011573.1 | ADP-ribosylation factor(Arf)/Arf-like (Arl) small GTPase family protein |
| XP_001025212.2 | hydroxymethylglutaryl-CoA lyase |
| XP_001028038.2 | peptidase family C54 protein |
| XP_001027702.1 | hypothetical protein |
| XP_001471498.1 | CDP-alcohol phosphatidyltransferase |
| XP_001008252.2 | peptide chain release factor 1 |
| XP_001007960.1 | tRNA pseudouridine synthase D |
| XP_001022259.2 | poly(A) polymerase, putative |
| XP_001030415.1 | bardet-biedl syndrome 1 family protein, putative |
| XP_001010647.2 | DNA polymerase |
| XP_001023472.1 | lipoate-protein ligase A |
| XP_001033039.2 | valyl-tRNA synthetase |
| XP_001014449.1 | pseudouridine synthase Rlu family protein |
| XP_001030185.2 | phospholipase D domain protein |
| XP_001026152.1 | cullin-4B protein |
| XP_001028110.1 | SIR2 family histone deacetylase, putative |
| XP_001022244.1 | hypothetical protein |
| XP_001024882.2 | AAA family ATPase |
| XP_001032778.2 | carrier protein |
| XP_012655768.1 | tRNA (guanine(37)-N1)-methyltransferase, putative |
| XP_976809.1 | U4/U6 small nuclear ribonucleoprotein Prp4 |
| XP_001018577.3 | hypothetical protein |
| XP_001023033.2 | coiled-coil and C2 domain protein 2A, putative |
| XP_001014247.1 | transcription initiation factor TFIID subunit 5 |
| XP_001022773.2 | ciliary basal body-associated B9 protein |
| XP_001016673.1 | phosphatidylserine synthase |
| XP_001031636.1 | mRNA capping enzyme |
| XP_001022635.1 | hypothetical protein |
| XP_001012813.1 | arginase |
| XP_001025563.2 | elongation factor Tu GTP-binding domain protein |
| XP_001022248.1 | tryptophanyl-tRNA synthetase protein |
| XP_001013747.3 | DNA polymerase II |
| XP_001022240.2 | ALG6, Alg8 glycosyltransferase family protein |
| XP_001023096.3 | copine protein |
| XP_001027834.1 | hypothetical protein |
| XP_001032724.1 | U3 small nucleolar RNA-associated protein, putative |
| XP_001014750.2 | DNA-directed RNA polymerase I protein |
| XP_001019364.1 | AAR2 protein |
| XP_001008721.3 | riboflavin kinase/FAD synthetase family protein |
| XP_001012670.1 | predicted protein |
| XP_012652651.1 | 4-hydroxybenzoate polyprenyltransferase |
| XP_001033394.1 | dihydrofolate reductase-thymidylate synthase |
| XP_001024120.1 | DEAD-box ATP-dependent RNA helicase |
| XP_001012102.2 | DEAD/DEAH-box helicase |
| XP_001016641.3 | KRI1 family carboxy-terminal protein |
| XP_001031723.1 | tetratricopeptide repeat protein |
| XP_001019344.2 | glucokinase |
| XP_001014682.2 | phosphoacetylglucosamine mutase |
| XP_001022465.2 | hypothetical protein |
| XP_001018226.3 | histidine phosphatase family (branch protein 1) |
| XP_001026360.1 | XPG amine-terminal domain protein |
| XP_001024178.3 | ribosomal RNA large subunit methyltransferase |
| XP_001020033.1 | 3-hydroxyacyl-CoA dehydrogenase |
| XP_001017111.2 | intraflagellar transport complex B protein 46 carboxy-terminal protein |
| XP_001026214.1 | amine-terminal domain cyclin |
| XP_001017169.1 | hypothetical protein |
| XP_001026746.1 | methyltransferase |
| XP_001026627.1 | ABC1 family protein |
| XP_012655920.1 | kinase domain protein |
| XP_001028137.1 | hypothetical protein |
| XP_001013224.2 | DNA mismatch repair MutS family DNA-binding domain protein, putative |
| XP_001020382.3 | tRNA methyltransferase complex GCD14 subunit |
| XP_001025504.1 | SMC family, carboxy-terminal domain protein |
| XP_001012837.1 | ubiquitin-activating enzyme E1 |
| XP_001015290.1 | ThiF family protein |

**Figure S1. Transmission electron micrographs of *P. bursaria*.** (A) *C. variabilis* (green arrow) locates beneath the cell cortex (black arrow). (B) *C. variabilis* is coated with PV membrane (red arrow) and occupies the position of trichocysts (yellow arrow). (C) PV-coated *C. variabilis* is dividing, and the division furrow is shown. (D) Newborn algae crumble away from the mother cell wall (blue arrow).

**Figure S2. Characteristics of the *P. bursaria* genome.** (A) The assembly of the *P. bursaria* genome, validated by TruSeq Synthetic Long-Read data. The assemblies from Illumina TruSeq Synthetic Long-Read were mapped to the *P. bursaria* genome, and 96.56% of the long-read data could be aligned. Contamination represents the assemblies of long-read data that cannot be mapped to the genome, which are likely derived from algae or bacteria. (B) The distribution of GC content of the assembled contigs in *P. bursaria*. (C) The Illumina sequencing depth of assembled contigs in *P. bursaria*.

**Figure S3. Comparative genomics of *P. bursaria* and other ciliates.** (A) GO enrichment analysis between *P. bursaria* and *P. caudatum*. GO terms related to oxygen binding and drug transporter activity are highlighted. (B) The number of globin genes in the MAC and the MIC of 11 ciliates. Ma, macronucleus and MIC, micronucleus. (C) BI and ML trees estimated from the globin genes of *P. bursaria* (PB, green), *P. caudatum* (PC, blue), *P. tetraurelia* (PT, black) and *O. trifallax* (OX, brown) under the evolutionary model ‘LG+G+F’. The solid black dots at nodes indicate Bayesian posterior probabilities (PPs) of 1.0 and bootstrap support (BS) values of 100%. The asterisks at nodes indicate bootstrap support values < 50%.

**Figure S4. GO enrichment analysis of protein-coding genes in *P. bursaria*.** (A) Biological process. (B) Molecular function. (C) Cellular component. The GO terms for which P < 0.05 (Pearson Chi-Square test) are shown.

**Figure S5. Summary of *P. bursaria* phenotype changes after RNAi experiment.** Three independent replicates, n = 3. *P < 0.05, **P < 0.01, ***P < 0.001, based on *t*-test.

**Figure S6. Microscope** **image of algae from crushed host cells.** (A) The control group. (B) The RNAi group at 72 hours.

**Figure S7. The co-expression network associated with GlnA in algae bearing and algae free *P. bursaria***. Node represents the gene and its size represents the P value of differential expression analysis (*t*-test). Lines in green colour show gene correlation in algae bearing *P. bursaria*, and lines in red colour show gene correlation in algae free *P. bursaria*. Pearson correlation value > 0.9.

**Figure S8. The structure of plasmids used for the RNAi experiment.** (A) The pMD19-T plasmid contains a T-cloning site, lacZ gene and KpnI and PstI restriction enzyme cutting sites. (B) The L4440 plasmid contains two T7 promoters and KpnI and PstI restriction enzyme cutting sites.

**Figure S1**

**Figure S2**

**Figure S3**

**Figure S4**

**Figure S5**

**Figure S6**

**Figure S7**

**Figure S8**
